# Translation, cross-cultural adaptation, validity and reliability of the inaugural Albanian Roland-Morris Disability Questionnaire in Albanian population with low back pain

**DOI:** 10.1371/journal.pone.0325777

**Published:** 2025-07-28

**Authors:** Orges Lena, Enkeleda Gjini, Jasemin Todri

**Affiliations:** 1 ÍTEM-Innovation in Manual and Physical Therapies-Research Group, Physiotherapy Department, UCAM Universidad Católica San Antonio de Murcia, Murcia, Spain; 2 Nursing Department, Health Science Faculty, Catholic University of Our Lady of Good Council, Tirana, Albania; Adnan Menderes Universitesi, TÜRKIYE

## Abstract

**Background context:**

To precisely evaluate the disability resulting from low back pain and the efficacy of interventions, it is essential to utilize a standardized instrument capable of measuring the patient’s condition changes over time. One of the most frequently utilized self-administered standardized questionnaires is the Roland Morris Disability Questionnaire (RMDQ).

**Purpose:**

To culturally adapt the RMDQ into Albanian and assess its psychometric properties within an Albanian population.

**Study design: Observational:**

Patient sample: The total sample consisted of 200 individuals diagnosed with various conditions including persistent low back pain, coxofemoral luxation, back trauma, lumbar fracture, and arthritis. Outcome measures: The RMQ consists of 24 individual items each presented as a single sentence. The maximum achievable score is 24, while the minimum is 0.

**Methods:**

The research procedure involved two phases: initially, the translation and cultural adaptation, and subsequently, the assessment of quality within a clinical investigation. Test-retest reliability at 7 days was evaluated in 200 participants.

**Results:**

Kappa statistics showed that Alb-RMDQ had acceptable item-by-item agreement for 11 items (k = 0.603–0.755) and the remaining 13 were moderate agreement (k = 0.464–0.595). The exploratory factor analysis (EFA) of Albanian version was used to determine this dimensionality. An initial EFA analysis with a principal component analysis using Varimax with Kaiser Normalization rotation method resulted in a 10-factor structure with a KMO = 0.465 and a Chi-square = 461.791, p = 0.000. The Interclass Correlation Coefficient (ICC) between the first and the second evaluation for each item performed with two mixed effect model was between 0.621–0.938 using an absolute agreement definition.

**Conclusions:**

The AlbRMDQ version of RMDQ has a good validity and reliability and may be an important outcome tool for the clinical and research purpose for the Albanian speaking population.

## Introduction

Low Back Pain (LBP) has long been a significant public health concern and a leading cause of work-related disability [1,2]. It is widely recognized as one of the most common painful conditions experienced throughout life, often prompting individuals to seek medical care [3,4]. The economic burden and disability levels associated with LBP are substantial, varying across countries due to differences in cultural norms, social structures, and perceptions of its causes and effects [5]. As a result, assessing the physical disability caused by LBP is a crucial patient-reported outcome that requires careful evaluation in both clinical practice and research. To ensure an accurate assessment of disability resulting from LBP and the effectiveness of interventions, it is essential to use reliable and standardized evaluation tools.

A practical approach for measuring activity limitations involves utilizing a patient self-reporting scale that consolidates relevant data. One of the most widely used self-administered standardized questionnaires for this purpose is the Roland Morris Disability Questionnaire (RMDQ). This tool is specifically designed to track changes in a patient’s condition over time, providing valuable insight into the impact of low back pain on daily activities and overall functionality [6–8].

To ensure accurate and meaningful assessment, the questionnaire should be available in the patient’s native language. Otherwise, physicians may need to translate or interpret the scale themselves, potentially affecting consistency and reliability [9]. The RMDQ was developed to measure the extent of disability affecting routine activities such as walking, sitting, dressing, sleeping, and work-related tasks [3]. It is widely recognized as a primary outcome measure in clinical trials for LBP and is frequently used in meta-analyses, cost-effectiveness studies, and multicenter research [10].

This scale aligns with the comprehensive biopsychosocial disability model outlined in the World Health Organization’s International Classification of Functioning, Disability, and Health (ICF). Guidelines based on cross-cultural adaptation research in medical, sociological, and psychological fields emphasize the importance of using self-report measures in diverse countries, cultures, and languages beyond their original development context [11,12]. This necessitates the application of specific translation and cultural adaptation techniques to ensure semantic, idiomatic, experiential, and conceptual equivalence between the original and translated versions of the questionnaire. Therefore, the aim of this study was to culturally adapt the RMDQ into Albanian and evaluate its psychometric properties within an Albanian population.

## Materials and methods

This study involved the cross-cultural adaptation, translation, validation, and reliability assessment of the RMDQ instrument in Albanian language. Ethical approval was obtained from the Ethics Committee of the Catholic University of Murcia (UCAM) under protocol No. CE052107 and the Ethics Committee of the Health Ministry and Social Defense in Tirana, Albania (protocol No. 147/35). The study was registered on ClinicalTrials.gov prior to data collection on 15/10/2019 (ID: NCT04131998). Participant enrollment began on 06/06/2021, and the study was completed on 31/07/2023.

The research procedure involved two phases: initially, the translation and cultural adaptation, and subsequently, the assessment of quality within a clinical investigation.

### Translation and cultural adaptation

The process of translation and cross-cultural adaptation followed the guidelines outlined by Beaton at al. 2000, describing a five steps procedure: translation, synthesis, reverse translation, expert committee review, and pretesting [12].

During the initial phase, two proficient Albanian translators independently translated the original RMDQ into Albanian.

One of the translators was informed with the RMDQ clinical content and spinal terminology. The second one had no medical background and was uninformed of the questionnaire concepts. Following this, the authors synthesized the two translations to produce the initial version in Albanian. Version one underwent to the back translation into English by two separate native English-speaking translators. They were unaware of the original English version of RMDQ and the study’s objectives.

An expert committee was convened to assess and evaluate all previously generated translations with the aim of refining them into the pre-final version of RMDQ in Albanian. The expert committee was formed by the translators, researchers, spinal specialists, and physiotherapists. Following the translation and cross-cultural adaptation process, a pretest was administered to ensure a level of quality in content validity. Fifty participants completed the last RMDQ version in Albanian and were asked for each item meaning.

The outcomes of the pretest were considered during the development of the final version of the Albanian RMDQ. As per construct validity analysis, the Consensus-Based Standards for the Selection of Health Measurement Instruments (COSMIN) recommends a sample size of 100–200 participants or approximately 5–10 participants per item, depending on the model’s complexity and the type of analysis conducted. For test–retest reliability, COSMIN advises a minimum of 50 participants, though a larger sample size is preferable, as it can provide more reliable estimates of the measurement’s stability over time [13].

The pre-test phase of the study involved careful evaluation of the translated RMDQ to ensure cultural and linguistic appropriateness. During this phase, a few minor linguistic ambiguities were identified in certain items and were refined to enhance clarity while preserving conceptual equivalence with the original questionnaire. However, no major structural or conceptual modifications were necessary. Given that the adjustments were minor and primarily related to wording for improved comprehension, the revised version was not re-administered to the pre-test participants. Instead, the study proceeded with the main validation phase using the final culturally adapted version. The literature acknowledges the recommendation to re-administer the questionnaire if significant changes are made [12]. However, in this case, the refinements did not alter the meaning of the items, and the final version was considered suitable for psychometric evaluation.

#### Participants.

The total sample consisted of 200 individuals diagnosed with various conditions including persistent low back pain, coxofemoral luxation, back trauma, lumbar fracture, and arthrosis.

The inclusion criteria for the RMDQ were determined to ensure the sample was representative of the population for which the adapted questionnaire was intended. Participants were included if they had been diagnosed with LBP and were able to understand and complete the questionnaire in Albanian. The sample also considered age and gender diversity to ensure a broad representation of individuals experiencing LBP.

Concretely, the inclusion criteria comprised native Albanian orthopedic patients experiencing articular and muscular pain, back pain, rheumatologic symptoms, functional imbalance, scoliosis, and musculoskeletal disorders, aged between 50 and 80 years. Participants with cognitive impairments or conditions that could interfere with their ability to accurately complete the RMDQ were excluded. The inclusion criteria were designed to ensure that the data collected would be valid and reflective of the target population’s experience with LBP. The Alb-RMDQ was administered at the Trauma and Orthopedic Specialty. Prior to data collection, informed written consent was obtained from all participants. Patients were instructed to complete the study questionnaires twice over a period of 7 days (test-retest). The initial data collection occurred during patients’ first hospital visits for their lower back complaints, while the second took place when patients attended their physiotherapeutic consultations.

#### Measurement tools.

The RMDQ consists of 24 individual items each presented as a single sentence.

Patients can assign one point to each item if they agree with the statement reflecting their condition on the day; otherwise, the item is scored as zero. Consequently, the maximum achievable score is 24, while the minimum is 0. A higher score suggests greater disability. The systematic application of the RMDQ can be considered in routine clinical practice, and there are no restrictions on its utilization [6].

#### Validity.

Validity was confirmed through content, construct, and criterion measures. An exploratory factor analysis (EFA) was conducted using principal components to ascertain the scale’s dimensions, considering criteria such as Bartlett’s test of sphericity and the Kaiser–Meyer–Olkin measure of sampling adequacy (eigenvalue >1, acceptable >0.5). The structure was assessed using orthogonal Varimax rotation, with the expectation that items would achieve a saturation level of 0.4 or higher in the one-dimensional scale.

Pain intensity was one of the factors considered in the validation process, but structural validity was primarily assessed through EFA, which considered multiple dimensions of disability beyond pain intensity.

#### Reliability.

Reliability was determined by assessing internal consistency. The RMDQ was applied to all the participants with a sample size n = 200.

The statistical analysis, employing Cronbach’s alpha coefficient, confirmed the acceptable range of this measurement (between 0.7 and 0.9) [14,15].

The first and second survey was administered to all the participants with a 7-day differences.

An intraclass correlation coefficient (ICC) value ranging between 0.7 and 0.8 is deemed acceptable, while a value exceeding 0.8 is considered excellent.

### Statistics

The measurement properties, including validity and reliability domains, of the Albanian RMDQ were examined based on the findings of the COSMIN Delphi study [13,16].

To assess the internal consistency of the RMDQ, Cronbach’s alpha value was computed. To evaluate reliability, the single-measure Intraclass Correlation Coefficient (ICC) was calculated for absolute agreement within a two-way mixed effect analysis of variance model.

Kappa statistic and ICC values of 0.80 or more were considered high, 0.60 to 0.80 to be acceptable, 0.41 to 0.6 moderate agreement, and 0.21 to 0.4 fair agreement [17].

A Cronbach’s alpha of 0.70 or higher indicates strong internal consistency among the items, supporting the construct validity as they measure the same underlying construct [18].

All statistical analyses were conducted using SPSS version 25.0 software, with significance set at p-values less than 0.05.

## Results

The mean age of the 200 participants in this study was 69.77 years (SD = 6.33). Males, who comprised 53% of the total sample (n = 106), had a mean age of 68.59 years (SD = 6.39), while females had a mean age of 71.09 years (SD = 6.00). Among diagnostic groups, participants with lumbar trauma had the highest mean age of 78.38 years (SD = 1.85, n = 18), whereas those with lumbar fractures had the lowest mean age of 68.07 years (SD = 6.33, n = 14). The most common diagnosis was nonspecific back pain (n = 86), with a mean age of 68.37 years (SD = 6.66). Participants diagnosed with lumbar trauma also reported the highest weekly physical activity, averaging 4.77 hours (SD = 0.646). Table 1 presents the characteristics of participants.

**Table 1:** Participant characteristics.

		Back Pain	Lumbar discal hernia	Lumbar trauma	Coxo-femoral Luxation	Lumbar fracture	Arthrosis
	N	86	5	18	5	14	72
**Gender**	Male	24	4	1	2	10	65
	Female	62	1	17	3	4	7
**Age**	Mean (SD)	68.37 (6.668)	74.20 (9.654)	78.388 (1.851)	72.00 (8.944)	68.071 (6.330)	69.138 (4.169)
**Pain duration day hours**		3.023 (0.547)	4.600 (0.894)	2.333 (0.970)	3.800 (1.095)	4.143 (0.534)	3.958 (0.910)
**Physical activity hours week**		4.697 (0.510)	3.200 (0.447)	4.777 (0.646)	3.600 (0.547)	3.928 (0.730)	3.486 (0.895)

SD = Standard deviation.

Kappa statistics showed that Albanian RMDQ (Alb-RMDQ) had acceptable item-by-item agreement for 11 items (k = 0.603–0.755) and the remaining 13 were moderate agreement (k = 0.464–0.595) (Table 2).

**Table 2:** Item by Item agreement of Alb-RMDQ.

Items	Kappa
Q1	0.631
Q2	0.552
Q3	0.518
Q4	0.557
Q5	0.464
Q6	0.540
Q7	0.661
Q8	0.565
Q9	0.755
Q10	0.701
Q11	0.579
Q12	0.623
Q13	0.691
Q14	0.753
Q15	0.489
Q16	0.571
Q17	0.595
Q18	0.603
Q19	0.588
Q20	0.644
Q21	0.543
Q22	0.520
Q23	0.683
Q24	0.668

Q = questions.

The exploratory factor analysis (EFA) of Albanian version was used to determine this dimensionality. An initial EFA analysis with a principal component analysis using Varimax with Kaiser Normalization rotation method resulted in a 10-factor structure with a KMO = 0.465 and a Chi-square = 461.791, p = 0.000. None of the items was removed (Table 3).

**Table 3:** Factor loading for 24 items of Alb-RMDQ.

Items	Factors									
	1	2	3	4	5	6	7	8	9	10
**Q15**	**0.675**									
**Q19**	**0.606**									
**Q16**		**0.716**								
**Q12**		**0.579**								
**Q5**		**0.402**								
**Q10**			**0.811**							
**Q9**			**0.803**							
**Q13**				**0.783**						
**Q4**				**0.652**						
**Q3**				**0.352**						
**Q2**					**0.687**					
**Q8**					**0.625**					
**Q1**						**0.755**				
**Q6**						**0.654**				
**Q24**						**0.188**				
**Q11**							**0.630**			
**Q17**							**0.454**			
**Q14**								**0.807**		
**Q7**								**0.645**		
**Q23**									**0.764**	
**Q22**									**−0.551**	
**Q20**									**0.442**	
**Q21**										**0.717**
**Q18**										**0.323**
The eigenvalue of each factor	**7.652**	**7.613**	**6.841**	**6.575**	**6.034**	**5.710**	**5.415**	**5.060**	**4.905**	**4.586**
% Explained variance of each factor	**7.652**	**15.265**	**22.106**	**28.682**	**34.716**	**40.426**	**45.841**	**50.901**	**55.806**	**60.392**

KMO = 0.465x2 = 461.791*KMO = Kaiser-Meyer-Olkin Measure of Sampling Adequacy; x2 = Bartlett’s Test of Sphericity tested with Chi-Square; *p = 0.000.

^a^Rotation converged in 18 iterations.

^b^Q = questions.

Extraction Method: Principal Component Analysis; Rotation Method: Varimax with Kaiser Normalization.

The Table 4 presents the fit indices only for the 2-factor solution in the Confirmatory Factor Analysis (CFA) of the Alb-RMDQ. The Chi-square (Χ²) value of 2.288 with 4 degrees of freedom (df) and a p-value of 0.683 suggest that the model does not significantly deviate from the observed data, indicating a good fit. The Root Mean Square Error of Approximation (RMSEA) was 0.000, with a 95% Confidence Interval (CI) ranging from −0.015 to 0.023, further supporting a perfect model fit. The Comparative Fit Index (CFI) of 1.000 and the Tucker-Lewis Index (TLI) of 1.233 exceed conventional thresholds (>0.95), suggesting an excellent fit. Overall, these results indicate that the 2-factor solution provides a well-fitting model for the Alb-RMDQ, supporting the structural validity of this measurement approach.

**Table 4:** Psychometric properties of Alb-RMDQ.

	Validity						Reliability			
	Internal consistency						Test-retest agreement			
*Items*	Scale statistics Mean (SD)	First Evaluation Mean (SD)	Second evaluation Mean (SD)	Inter-iterm Covariances	Inter-iterm Correlations	Crombach’s Alpha	ICC	95% CI	F	*p*
***Q1***	1.57 (0.747)	0.84 (0.368)	0.73 (0.445)	0.112	0.687	0.806	0.789	(0.700-0.849)	5.145	0
***Q2***	1.78 (0.597)	0.91 (0.294)	0.87 (0.337)	0.078	0.787	0.876	0.874	(0.833-0.905)	8.095	0
***Q3***	1.82 (0.492)	0.96 (0.208)	0.86 (0.348)	0.039	0.538	0.643	0.621	(0.482-0.720)	2.801	0
***Q4***	1,63 (0.719)	0.85 (0.363)	0.78 (0.415)	0.106	0.706	0.824	0.818	(0.756-0.863)	5.666	0
***Q5***	0.21 (0.543)	0.14 (0.348)	0.07 (0.247)	0.056	0.653	0.763	0.750	(0.658-0.815)	4.223	0
***Q6***	1.92 (0.346)	0.98 (0.157)	0.95 (0.218)	0.024	0.698	0.796	0.792	(0.725-0.843)	4.897	0
***Q7***	1.87 (0.463)	0.94 (0.238)	0.93 (0.256)	0.046	0.756	0.86	0.860	(0.815-0.894)	7.127	0
***Q8***	1.46 (0.856)	0.76 (0.428)	0.71 (0.457)	0.17	0.869	0.929	0.925	(0.898-0.945)	14.022	0
***Q9***	1.74 (0.620)	0.89 (0.314)	0.85 (0.358)	0.079	0.703	0.821	0.818	(0.760-0.863)	5.591	0
***Q10***	1.66 (0.698)	0.85 (0.358)	0.81 (0.393)	0.102	0.725	0.838	0.836	(0.783-0.876)	6.179	0
***Q11***	1.82 (0.541)	0.93 (0.264)	0.89 (0.314)	0.062	0.749	0.849	0.847	(0.796-0.884)	6.642	0
***Q12***	1.71 (0.640)	0.88 (0.326)	0.83 (0.381)	0.079	0.64	0.775	0.770	(0.695-0.826)	4.436	0
***Q13***	1.91 (0.378)	0.97 (0.184)	0.95 (0.229)	0.028	0.67	0.791	0.790	(0.723-0.841)	4.794	0
***Q14***	1.81 (0.555)	0.92 (0.280)	0.89 (0.314)	0.066	0.752	0.855	0.854	(0.808-0.890)	6.918	0
***Q15***	0.80 (0.949)	0.44 (0.497)	0.37 (0.484)	0.21	0.873	0.932	0.928	(0.901-0.947)	14.759	0
***Q16***	1.73 (0.599)	0.90 (0.301)	0.83 (0.377)	0.063	0.559	0.706	0.697	(0.597-0.772)	3.398	0
***Q17***	1.50 (0.821)	0.73 (0.448)	0.77 (0.422)	0.147	0.781	0.876	0.874	(0.833-0.905)	8.074	0
***Q18***	1.81 (0.528)	0.92 (0.289)	0.89 (0.314)	0.051	0.58	0.731	0.731	(0.645-0.796)	3.724	0
***Q19***	0.61 (0.895)	0.33 (0.470)	0.29 (0.453)	0.188	0.886	0.939	0.938	(0.917-0.953)	16.486	0
***Q20***	1.62 (0.761)	0.82 (0.389)	0.80 (0.401)	0.134	0.856	0.922	0.922	(0.897-0.941)	12.882	0
***Q21***	1.92 (0.36)	0.98 (0.157)	0.95 (0.218)	0.024	0.698	0.796	0.792	(0.725-0.843)	4.897	0
***Q22***	0.97 (0.958)	0.50 (0.501)	0.47 (0.500)	0.208	0.831	0.908	0.907	(0.878-0.930)	10.83	0
***Q23***	1.68 (0.671)	0.85 (0.354)	0.83 (0.377)	0.091	0.683	0.811	0.810	(0.749-0.856)	5.278	0
***Q24***	0.20 (0.564)	0.10 (0.301)	0.10 (0.294)	0.071	0.801	0.890	0.890	(0.855-0.917)	9.063	0

Table 5 shows the psychometric properties of Alb-RMDQ were Crombach’s Alpha for each item evaluated at baseline and after 7 days ranges between 0.643–0.939. The inter-item correlations range from 0.538–0.886. The ICC between the first and the second evaluation for each item performed with two mixed effect model was between 0.621–0.938 using an absolute agreement definition. ICC indicated a good to excellent reliability and reproducibility of Alb-RMDQ with a p = 0.000 in all items.

**Table 5:** Summary of CFA fit indexes for the 2-factor solution.

Model	Χ²	df	P	RMSEA	95% CI	CFI	TLI
2-Factor solution	2.288	4	0.683	0	−0.015–0.023	1	1.233

RMSEA = root mean square error of approximation, CI = confidence interval, CFI = comparative fit index, TLI = Tucker–Lewis’s index.

## Discussion

This cross- cultural study presents the outcomes of the first translated-validated Albanian RMDQ. The RMDQ demonstrates strong responsiveness to clinical changes, making it useful for monitoring treatment outcomes in both clinical and research settings [19,20]. Its validity and reliability have been established across various populations, reinforcing its applicability in diverse clinical contexts [21,22]. However, the RMDQ also has notable limitations. It exhibits a ceiling effect, meaning that it may not effectively differentiate between individuals with severe disability, as patients with significant functional impairment often score at the upper limit of the scale [23]. Furthermore, the questionnaire primarily focuses on physical disability and does not comprehensively assess other important dimensions of low back pain, such as psychological and social factors, which are increasingly recognized as crucial in pain management [21,22,24]. Compared to more comprehensive tools, such as the Oswestry Disability Index (ODI), the RMDQ may have lower sensitivity in detecting small functional improvements in certain patient groups [25]. Despite these limitations, the RMDQ remains a valuable tool due to its practicality, ease of administration, and responsiveness, particularly in mild-to-moderate disability cases.

The original structure with 24 items were adapted and validated into Albanian. The Alb-RMDQ was easy to understand and to administer in 200 participants. No language difficulty was shown. None of participants referred difficulty or any problem completing the questionnaire. The same results were shown in other studies where a good comprehension and acceptability was demonstrated [15,26].

The gender distribution curiously was equal in this cross-cultural study. The mean age of all participants was 69.76 with a SD of 6.33. The minimum was 55 years old, and the maximum was 88 years old. This fact maybe contributed to the pain intensity or diagnosis.

Referring to each item (α = 0.643–0.939) the Alb-RMDQ demonstrated good internal consistency, conforming to other studies which evaluated the other translated-adapted versions of RMDQ [27,28].

Test-retest values (ICC = 0.621–0.938) indicated that Alb-RMDQ had high short-term results, is reliable, reproducible tool and with good agreement with English RMDQ in Albanian population with LBP. The duration of test-retest between 3–7 days can affect the study results. This fact can be related with the same clinical status of participants during the first week of evaluation. Concretely, in the absence of a targeted intervention, patients with chronic pain are unlikely to experience significant changes in their clinical status during this period.

No dropped-out participants were observed. Comparing these results with the study of Lee at al. 2011, where 81 participants were missing in the retest questionnaire, it can be said that maybe the 1–2 weeks interval can be longer time to track the same participants. Also, the recruiting retest results by mail can affected the participants number, as referred Lee et al 2011 [29].

The Albanian version of the RMDQ had no floor/ceiling effects, as no patient achieved either of the maximum or the minimum possible scores. The Effect Size (ES) and Standardized Response Mean (SRM) values for items in the Alb-RMDQ showed varying responsiveness to changes in disability. The Q3 item demonstrated the highest responsiveness (ES = 0.2032, SRM = 0.2032), while the Q24 item (ES = 0.0000, SRM = 0.0000) showed no measurable change. Some items, such as Q7 and Q19, exhibited low responsiveness, indicating limited effectiveness in tracking changes. A study by Macedo et al. (2011) compared the responsiveness of different RMDQ versions and found that the 18-item versions were more responsive than the 24- and 11-item versions. This general finding of moderate responsiveness aligned with our study’s observation of varying item responsiveness [30]. Given the lack of publicly available item-specific responsiveness data, it is difficult to directly compare individual items like Q3, Q24, Q7, and Q19 with those in other studies. Future research that reports item-level responsiveness metrics would facilitate more precise comparisons and enhance the understanding of each item’s effectiveness in detecting changes in disability.

Regarding the external validity of the Alb-RMDQ, it demonstrated a positive correlation with the Albanian Oswestry Disability Index (ODI-A) [31] (r = 0.232, p = 0.001) and pain duration (r = 0.174, p = 0.014). However, its association with the Visual Analogue Scale (VAS) was less evident, showing no statistically significant correlation (Table 6).

**Table 6:** Pearson’s correlation coefficients of the Alb-RMDQ with Albanian Oswestry Disability Index (ODI-A), Visual Analogue Scale (VAS) and pain duration.

		Alb-RMDQ	ODI-A	VAS	Pain Duration hours per day
**Alb-RMDQ**	**Pearson Correlation**		.232**	0.049	.174*
	**Sig. (2-tailed)**		0.001	0.487	0.014
**ODI-A**	**Pearson Correlation**	.232**		.304**	.712**
	**Sig. (2-tailed)**	0.001		0	0
**VAS**	**Pearson Correlation**	0.049	.304**		.460**
	**Sig. (2-tailed)**	0.487	0		0
**Pain Duration (hours per day)**	**Pearson Correlation**	.174*	.712**	.460**	
	**Sig. (2-tailed)**	0.014	0	0	

*Correlation is significant at the 0.05 level (2-tailed).

**Correlation is significant at the 0.01 level (2-tailed).

As per acceptability analysis, patients did not encounter any difficulty in completing the questionnaire, with an average completion time of 5 minutes. The questionnaire demonstrated 100% acceptability, indicating high satisfaction. No participants declined to complete it, and many appreciated that it addressed aspects of their condition often overlooked during routine consultations for LBP. The process was found to be straightforward and efficient.

This study’s findings indicate that the translation of the RMDQ into Albanian was effective, preserving the psychometric characteristics of the original version.

As far as the authors are aware, this Albanian version of the RMDQ represents the inaugural validation of a specific condition outcome measure for LBP within an Albanian population.

Creating and validating multiple-language versions of established questionnaires is crucial for standardizing outcome assessments and enhancing the statistical robustness of clinical studies in Albania.

Hence, the Albanian iteration of the RMDQ emerges as a dependable and valid tool for evaluating functional status among individuals with LBP. Consequently, we advocate for the adoption of this Albanian version of the RMDQ in forthcoming clinical investigations conducted in Albania.

This study had some limitation as was the first validation study in Albanian for the RMDQ which consisted in a lack of confrontable literature and same language feedback results.

The seven days retest can affect the results because of the closer time of data collection and the same participant’s condition.

Construct validity of Alb-RMDQ was evaluated specifically for the single dimension it aims to measure, which is pain intensity.

## Conclusion

The Alb-RMDQ version of RMDQ has a good validity and reliability and may be an important outcome tool for the clinical and research purpose for the Albanian speaking population.
