# Correction: Translation, cross-cultural adaptation, validity and reliability of the inaugural Albanian Roland-Morris Disability Questionnaire in Albanian population with low back pain

**DOI:** 10.1371/journal.pone.0350327

**Published:** 2026-05-28

**Authors:** The PLOS One Editors

## Notice of Republication

This article [1] was republished on April 17, 2026, to address an issue identified post-publication. An updated version of S1 File is provided with this notice. Please download this article again to view the correct version.

A new Supporting Information file (S2 File) containing the Albanian language version of the questionnaire is also provided with this notice.

In the Study design: Observational subsection of the Abstract, there is an error in the first sentence. The correct sentence is: Patient sample: The total sample consisted of 200 individuals diagnosed with various conditions including persistent low back pain, coxofemoral luxation, back trauma, lumbar fracture, and arthrosis.

The article’s Data Availability statement is updated to: All relevant data are within the paper and its Supporting Information files.

## Supporting information

S1 File Underlying data. (XLSX)

S2 File Albanian-language version of the Roland Morris Disability Questionnaire (RMDQ). (PDF)
