# Supplementary material for: Correction: Translation, cross-cultural adaptation, validity and reliability of the inaugural Albanian Roland-Morris Disability Questionnaire in Albanian population with low back pain
Source: PLoS One. 2026 May 28;21(5):e0350327. doi: 10.1371/journal.pone.0350327 (PMC13218512; doi:10.1371/journal.pone.0350327)
Supplement: S2 File — (PDF) [file pone.0350327.s002.pdf]

PYETESORI I ROLAND MORRIS PER DHIMBJEN E SHPINES DHE AFTESIVE TE KUFIZUARA (RMQ)

EMER, MBIEMER ____________________ KODIFIKIMI_______________ DATA________________________-

Kur ju dhemb shpina, mund te rezultoje e veshtire te realizohen disa gjera te zakonshme. Kjo liste permban shprehi te perdorura nga individe te cilet pershkruajne veten kur kane dhimbje shpine. Kur ti lexoni, mund te ndesheni me disa fjali te cilat ju pershkruajne me se miri sot. Kur te lexoni fjalite te cilat ju pershkruajne me konkretisht, vendosni nje shenje. Nese fjalia me poshte nuk peshkruan gjendjen tuaj, lini hapesire boshe dhe vazhdoni tek tjetra. Mos harroni, shenoni vetem fjaline e cila ju pershkruan sot.
Pikezimi: piket jane numri total i fjalive te shenuara d.m.th. nga minimumi 0 deri ne maksimum 24.

1. Unë qëndroj në shtëpi shumicën e kohës për shkak të shpinës.
2. Ndryshoj pozicionin shpesh për të rehatuar shpinën time.
3. Unë eci më ngadalë se zakonisht për shkak të shpinës.
4. Për shkak të shpinës sime nuk po bëj asnjë nga punët që bëj zakonisht nëpër shtëpi.
5. Për shkak të shpinës, unë mbahem tek parmaku për të ngritur lart.
6. Për shkak të shpinës, unë shtrihem për të pushuar më shpesh.
7. Për shkak të shpinës, duhet të mbahem diku për tu ngritur nga karrigia.
8. Për shkak të shpinës, unë përpiqem t'i them të tjerëve të bëjnë gjëra për mua.
9. Unë vishem më ngadalë se zakonisht për shkak të shpinës.
10. Për shkak të shpinës, unë qëndroj në këmbë vetëm për periudha të shkurtra kohe.
11. Për shkak të shpinës, përpiqem të mos përkulem ose gjunjëzohem.
12. E kam të vështirë të ngrihem nga një karrige për shkak të shpinës.
13. Më dhemb shpina pothuajse gjatë gjithë kohës.
14. E kam të vështirë të lëviz në shtrat për shkak të shpinës.
15. Nuk kam oreks shumë të mirë për shkak të dhimbjes në shpinë.
16. Kam probleme në veshujen e çorapëve për shkak të dhimbjes së shpinës.
17. Unë vetëm eci me distanca të shkurtra për shkak të shpinës.
18. Unë e bëj gjumin më pak të rehatshem për shkak të shpinës.
19. Për shkak të dhimbjes në shpinë, më ndihmon dikush tjetër të vishem.
20. Unë qëndroj ulur për pjesën më të madhe të ditës për shkak të shpinës.
21. Unë shmang punët e rënda përreth shtëpisë për shkak të shpinës.
22. Për shkak të dhimbjes në shpinë, unë jam me njerëzit më i irrituar dhe më me humor të keq se zakonisht.
23. Për shkak të shpinës, unë i ngjis shkallët më ngadalë se zakonisht.
24. Unë qëndroj në shtrat shumicën e kohës për shkak të shpinës.

Roland-Morris Low Back Pain
And Disability Questionnaire (RMQ)

Source: Stratford PW, Binkley J, Solomon P, Finch E, Gill C, Moreland J. Defining the minimum level of detectable change for the Roland-Morris questionnaire. *Phys Ther*. 1996 Apr;76(4):359-65; discussion 66-8.

The Roland-Morris Questionnaire (RMQ) is a self-administered disability measure in which greater levels of disability are reflected by higher numbers on a 24-point scale. The RMQ has been shown to yield reliable measurements, which are valid for inferring the level of disability, and to be sensitive to change over time for groups of patients with low back pain.

Scoring instructions

The patient is instructed to put a mark next to each appropriate statement. Add up the total number of marked statements to get a patient's score.

Interpretation of scores

Roland and Morris did not provide descriptions of the varying degrees of disability (eg, 40%-60% is severe disability). Clinical improvement over time can be graded based on the analysis of serial questionnaire scores. If, for example, at the beginning of treatment, a patient's score was 12 and, at the conclusion of treatment, their score was 2 (10 points of improvement), we would calculate an 83% (10/12 x 100) improvement.

Roland-Morris Low Back Pain and Disability Questionnaire (RMQ)

Instructions

Patient name:__________________________________ File #:____________ Date:_____________

Please read instructions: When your back hurts, you may find it difficult to do some of the things you normally do. Mark only the sentences that describe you today.

- ☐ I stay at home most of the time because of my back.
- ☐ I change position frequently to try to get my back comfortable.
- ☐ I walk more slowly than usual because of my back.
- ☐ Because of my back, I am not doing any jobs that I usually do around the house.
- ☐ Because of my back, I use a handrail to get upstairs.
- ☐ Because of my back, I lie down to rest more often.
- ☐ Because of my back, I have to hold on to something to get out of an easy chair.
- ☐ Because of my back, I try to get other people to do things for me.
- ☐ I get dressed more slowly than usual because of my back.
- ☐ I only stand up for short periods of time because of my back.
- ☐ Because of my back, I try not to bend or kneel down.
- ☐ I find it difficult to get out of a chair because of my back.
- ☐ My back is painful almost all of the time.
- ☐ I find it difficult to turn over in bed because of my back.
- ☐ My appetite is not very good because of my back.
- ☐ I have trouble putting on my socks (or stockings) because of the pain in my back.
- ☐ I can only walk short distances because of my back pain.
- ☐ I sleep less well because of my back.
- ☐ Because of my back pain, I get dressed with the help of someone else.
- ☐ I sit down for most of the day because of my back.
- ☐ I avoid heavy jobs around the house because of my back.
- ☐ Because of back pain, I am more irritable and bad tempered with people than usual.
- ☐ Because of my back, I go upstairs more slowly than usual.
- ☐ I stay in bed most of the time because of my back.
